# Divergent Assembly of Bacteria and Fungi During Saline–Alkali Wetland Degradation

**DOI:** 10.3390/biology15010061

**Published:** 2025-12-29

**Authors:** Junnan Ding, Yingjian Wang, Shaopeng Yu

**Affiliations:** 1 Heilongjiang Province Key Laboratory of Cold Region Wetland Ecology and Environment Research, Harbin University, Harbin 150086, China; ding.junnan@163.com; 2 School of Chemistry and Molecular Bioscience, Queensland University, Brisbane, QLD 4072, Australia; wyingjian12@gmail.com

**Keywords:** saline–alkali wetland degradation, bacterial and fungal communities, community assembly, environmental filtering, stochastic processes

## Abstract

To clarify microbial assembly during saline–alkali wetland degradation, we analyzed bacterial (16S rRNA) and fungal (ITS) communities across four habitats: pristine wetland (PW), transitional meadow wetland (TMW), halophytic herbaceous community (HHC), and converted farmland (CF). Soil water content collapsed from PW (42.22%) to ≤18.40% elsewhere, and soils were alkaline with pH highest in HHC (10.08). Nutrient pools and enzyme activities were highest in PW (SOC 35.03 g kg^−1^; URE 142.58 mg g^−1^; SUC 527.83 mg g^−1^) but declined sharply under natural degradation, reaching minima in HHC (SOC 8.02 g kg^−1^). ACP and CAT were also lowest in HHC. Bacterial communities were dominated by Actinomycetota and Pseudomonadota, with Acidobacteriota and Bacillota enriched in CF. Bacterial diversity peaked in CF, whereas fungal richness was highest in CF and Shannon diversity peaked in TMW. Ordination and redundancy analyses indicated stronger edaphic control on bacteria than fungi, with pH, SOC, and moisture as key drivers. Null-model analyses showed bacterial assembly shifted toward deterministic selection under saline–alkali stress and agricultural conversion, whereas fungal assembly remained predominantly stochastic. Co-occurrence networks further suggested higher bacterial vulnerability under extreme degradation but comparatively higher fungal robustness. Overall, bacteria and fungi follow divergent assembly rules during saline–alkali wetland degradation.

## Simple Summary

Saline–alkali wetlands in Northeast China are shrinking because water levels are declining and some wetlands are drained and converted to cropland, which can reduce soil fertility and weaken ecosystem functions. We compared soil microorganisms across four habitats: intact wetland, a partially dried meadow wetland, a severely salt-affected grassland with salt-tolerant plants, and cropland converted directly from wetland. We measured soil moisture, alkalinity, nutrients, and soil enzyme activities (chemicals made by soil life that help recycle nutrients), and used DNA sequencing to describe bacteria and fungi. Drying caused a sharp drop in soil moisture, and the most salt-affected habitat had the highest alkalinity and the lowest nutrient levels and enzyme activities. Bacteria tracked these soil changes closely and were increasingly shaped by harsh environmental filtering, meaning only tolerant types persisted under extreme stress and farming disturbance. Fungi were less tightly linked to bulk soil conditions, were more influenced by chance and small habitat differences, and kept relatively stable links under stress. These results highlight an early restoration window: restoring water connectivity soon after drying begins may prevent severe degradation. Where soils are already highly alkaline, lowering alkalinity, rebuilding nutrients, and supporting vegetation recovery can aid ecosystem recovery.

## 1. Introduction

Saline–alkali wetland degradation has become a major ecological concern in the Songnen Plain of Northeast China. In this region, wetlands are threatened by both natural hydrological decline and intensive human activities, including reclamation and agricultural expansion, leading to wetland loss and secondary soil salinization and alkalinization [1]. These changes modify soil moisture, pH, salinity, and nutrient availability, which in turn drive vegetation shifts and weaken biodiversity and ecosystem functioning [2]. As one of the world’s largest soda-type saline–alkali regions, the Songnen Plain provides a distinctive setting for examining how soil environments and biotic communities reorganize during wetland degradation [3].

Soil microbial communities play essential roles in wetland ecosystems by regulating organic matter decomposition, nutrient cycling, and plant–soil feedback [4]. In saline–alkali wetlands, soil pH, salinity, and nutrient status can act as strong environmental filters that reshape microbial diversity and community composition [5]. Increasing stress commonly favors tolerant taxa and may alter microbial association patterns, with implications for carbon, nitrogen, and phosphorus cycling as well as ecosystem resilience [6]. At the same time, biotic processes, including plant-mediated substrate supply and microbial interactions, can also influence community structure, particularly during vegetation replacement and progressive habitat stress [7].

A central question in microbial ecology is how deterministic processes, such as environmental filtering, and stochastic processes, such as dispersal limitation and ecological drift, jointly govern community assembly along degradation gradients [8]. Many studies suggest that deterministic selection strengthens under harsher conditions [9]. However, bacteria and fungi may respond differently because they differ in life-history strategies, resource acquisition, and dispersal traits. In addition, microbial communities can modify their environment through biochemical transformations, generating feedback that influences ecosystem stability [10]. Therefore, comparing assembly mechanisms between bacteria and fungi, and linking these mechanisms to community association structure, is important for understanding microbial resilience during degradation.

The Songnen Plain contains a clear sequence from intact wetlands to transitional meadow wetlands and severely salinized halophytic communities, while large areas have also been converted directly from wetlands to cropland [11]. This landscape, therefore, represents two contrasting trajectories: natural regressive succession driven by hydrological disconnection and anthropogenic conversion driven by cultivation. Previous work has identified soil pH and salinity, nutrient availability, and plant-related factors as important determinants of microbial diversity and community structure across degradation stages [12,13]. Nevertheless, it remains unclear whether bacteria and fungi follow similar assembly rules under these contrasting trajectories, and whether changes in abiotic filtering and microbial associations lead to consistent shifts in community stability during rapid land-use change [14,15].

In this study, we investigated bacterial and fungal communities across four representative habitats in the Songnen Plain, including pristine wetland, transitional meadow wetland, halophytic herbaceous community, and converted farmland. We combined soil physicochemical properties and enzyme activities with high-throughput sequencing to characterize community variation and infer assembly processes, and we evaluated co-occurrence network topology and stability to connect community organization with potential resilience under stress. We tested the following hypotheses. First, bacterial communities become increasingly shaped by deterministic selection as saline–alkali stress intensifies and as land use is converted. Second, fungal communities exhibit weaker constraints from bulk soil properties and remain predominantly governed by stochastic processes across habitats. Third, extreme saline–alkali stress is associated with reduced stability in bacterial association networks, whereas fungal networks exhibit comparatively higher robustness under stress [16].

## 2. Materials and Methods

### 2.1. Site Information

**Figure 1:**
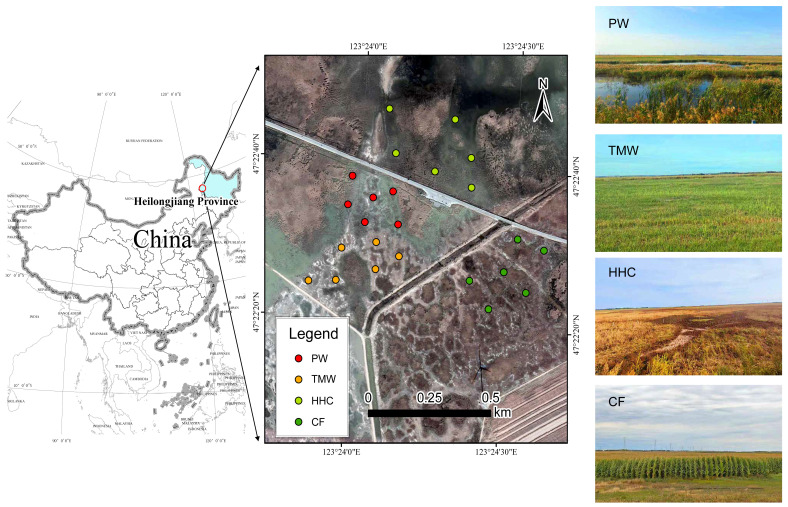
Location map of the study area in the Songnen Plain. Abbreviations: PW, pristine wetland; TMW, transitional meadow wetland; HHC, halophytic herbaceous community; CF, converted farmland (maize fields reclaimed from former wetlands).

The field investigation was conducted in the Halahai Provincial Nature Reserve and its surrounding areas, located within the Songnen Plain, Heilongjiang Province, Northeast China (47°20′ N, 124°10′ E). This region is characterized by a temperate continental monsoon climate, with a mean annual temperature of approximately 3.2 °C and an annual precipitation of about 400 mm. The dominant soil types are saline meadow soils and alkaline meadow soils, shaped by long-term hydrological fluctuations and strong salinization processes. To investigate the impacts of wetland degradation and agricultural conversion, four representative habitat types were delineated. These sites represent two distinct trajectories of land-use change: natural regressive succession driven by water retreat and anthropogenic reclamation driven by cultivation. (1) PW (pristine wetland) represents the reference state of the healthy ecosystem. This area is seasonally or permanently flooded and dominated by dense stands of *Phragmites australis* and *Aster* spp., maintaining high soil moisture, relatively low salinity, and well-developed organic horizons with minimal human disturbance. (2) TMW (transitional meadow wetland) represents a mildly degraded stage. Due to the gradual decline in groundwater levels over the past decades, this site has shifted from a marsh to a meadow ecosystem. It is characterized by mixed communities of *Leymus chinensis*, *Carex* spp., and *Calamagrostis epigejos*, subject to seasonal drought and light grazing disturbance. (3) HHC (halophytic herbaceous community) represents a severely degraded and salinized stage. Driven by long-term hydrological disconnection and intense surface evaporation, soil salinity has accumulated significantly. The original vegetation has been replaced by halophytic communities dominated by *Suaeda glauca*, with visible salt crusts on the soil surface, indicating strong environmental filtering. (4) CF (converted farmland) represents the agricultural reclamation state. Crucially, these sites were reclaimed directly from the pristine Phragmites wetland (similar to PW) approximately 10 years ago, rather than from already degraded saline soils. The land has undergone continuous drainage, tillage, and fertilization for maize (*Zea mays*) cultivation, which has fundamentally altered the original soil structure and hydrological regime. This four-stage framework provides a clear ecological context for evaluating vegetation dynamics and soil microbial responses along both natural degradation and agricultural conversion gradients. The spatial distribution of the study area and these four vegetation types is presented in Figure 1.

### 2.2. Sample Collection

Sampling was conducted from 11 to 12 September 2025. Each of the four vegetation types (PW, TMW, HHC, and CF) comprised six replicate sampling locations. Within each vegetation type, replicate locations were separated by at least 30 m to reduce spatial autocorrelation. Replicate locations were randomly selected within each habitat patch. Within each replicate location, multiple soil cores were collected following an envelope-pattern layout and pooled to form one composite sample to reduce microsite effects and improve representativeness. At each location, soil cores were collected at a standardized depth of 0–20 cm. Bulk soil was targeted in this study, and rhizosphere soil was not sampled separately. During sampling, we avoided visibly root-dense zones as much as possible, and visible roots, litter, stones, and other debris were carefully removed prior to homogenization. Soil from each replicate location was homogenized to form a composite sample, sealed in labeled bags, kept at approximately 4 °C during transport, and promptly delivered to the laboratory. Upon arrival, approximately 10 g of fresh soil from each composite sample was stored at −80 °C for microbial DNA extraction, while the remaining portion was air-dried and processed for physicochemical analyses.

### 2.3. Analysis of Soil Physicochemical Properties

Soil samples were subjected to measurement of multiple physicochemical parameters using established laboratory methods. Fresh soil samples were weighed, oven-dried at 105 °C to constant weight, and soil water content (SWC) was calculated as the percentage loss in mass relative to the fresh weight [17]. Soil pH was measured potentiometrically using a calibrated glass-electrode pH meter in a 1:2.5 (*w*/*v*) soil-to-deionized water suspension. The mixture was stirred and allowed to equilibrate before measurement [18]. Soil organic carbon (SOC) was determined by the Walkley–Black wet oxidation method, soil samples were digested with potassium dichromate and sulfuric acid, and the remaining dichromate was titrated with ferrous sulfate [19]. Total nitrogen (TN) was measured using the Kjeldahl digestion method. Soil samples were digested with concentrated sulfuric acid in the presence of a catalyst, converting organic nitrogen to ammonium, which was then quantified by distillation and titration [20]. Total phosphorus (TP) was determined by acid digestion with a mixture of perchloric and sulfuric acids, followed by colorimetric quantification using the molybdenum blue method [21]. Alkali-hydrolyzable nitrogen (AN) was measured using the alkali-hydrolysis diffusion method. Soil was treated with sodium hydroxide, and the released ammonia was collected in a boric acid trap and titrated [22]. Available phosphorus (AP) was extracted with 0.5 M NaHCO_3_ (pH 8.5) and quantified by the molybdenum blue colorimetric method [23]. Catalase activity (CAT) was measured by incubating soil with hydrogen peroxide and titrating the residual H_2_O_2_ with potassium permanganate. Results were expressed as mg H_2_O_2_ decomposed per g soil [24]. Urease activity (URE) was determined using a colorimetric microplate method. Soil was incubated with a urea solution, and the ammonium produced was measured colorimetrically, typically using indophenol blue or Nessler’s reagent, and expressed as mg NH_4_^+^-N per g soil per hour [25]. Acid phosphatase activity (ACP) was assayed using p-nitrophenyl phosphate as a substrate. Soil was incubated with the substrate, and the released p-nitrophenol was measured spectrophotometrically at 400 nm. Results were expressed as mg p-nitrophenol per g soil per hour [26]. Sucrase activity (SUC) was measured by incubating soil with sucrose solution, followed by colorimetric determination of the glucose produced, typically using 3,5-dinitrosalicylic acid (DNS) reagent. Results were expressed as mg glucose per g soil per hour [27].

### 2.4. DNA Extraction and High-Throughput 16S rRNA Gene Paired-End Sequencing

Genomic DNA was extracted from 0.5 g of fresh soil using the Omega E.Z.N.A.^®^ Soil DNA Kit (Omega Bio-Tek, Norcross, GA, USA) according to the manufacturer’s instructions. This kit employs bead beating in Disruptor Tubes preloaded with glass beads and an inhibitor-removal reagent (cHTR) to effectively eliminate humic substances and other PCR inhibitors, ensuring high-quality DNA suitable for downstream amplification and sequencing. The integrity and purity of the extracted DNA were verified by 1% agarose gel electrophoresis, which showed intact high-molecular-weight bands with minimal smearing, and by spectrophotometric assessment (A_260_/A_280_ and A_260_/A_230_ ratios). Bacterial community composition was characterized by amplifying the V3–V4 hypervariable region of the 16S rRNA gene using primers 515F (5′-GTGCCAGCMGCCGCGGTAA-3′) and 907R (5′-CCGTCAATTCMTTTRAGTTT-3′), while fungal communities were assessed by amplifying the ITS2 region using primers ITS3 (5′-GCATCGATGAAGAACGCAGC-3′) and ITS4 (5′-TCCTCCGCTTATTGATATGC-3′). PCR amplification was performed in two stages: the first targeted the respective gene regions, and the second appended sample-specific barcodes and Illumina adapters. The PCR thermal profile was as follows: initial denaturation at 95 °C for 3 min, 25 cycles of 95 °C for 30 s, 55 °C for 30 s, and 72 °C for 45 s, and a final extension at 72 °C for 10 min. Amplicons were purified using a commercial PCR cleanup kit, quantified with a QuantiFluor^®^-ST fluorometer (Promega, Madison, WI, USA), normalized to equimolar concentrations, pooled, and subjected to paired-end sequencing (2 × 250 bp) on the Illumina HiSeq 2500 platform (Shanghai Meiji Biotechnology Co., Ltd., Shanghai, China). Both bacterial and fungal sequencing data were subsequently used for downstream bioinformatics and ecological network analyses. All raw sequencing data have been deposited in the NCBI Sequence Read Archive (SRA) under BioProject accession numbers PRJNA1354685 and PRJNA1354678, respectively.

### 2.5. Microbial Community Statistical and Bioinformatic Analyses

Data normality and homogeneity of variance were first assessed using the Shapiro–Wilk and Levene’s tests to ensure the validity of parametric analyses [28]. When these assumptions were satisfied, one-way ANOVA (two-tailed, α = 0.05) was performed in IBM SPSS Statistics v22.0 (IBM Corp., Armonk, NY, USA), with Tukey’s HSD for post hoc comparisons; for heteroscedastic data, Welch’s ANOVA with Games–Howell was used [29]. Bivariate associations between environmental variables and dominant taxa were evaluated using Spearman’s rank correlation (ρ) in R v3.3.1 (R Foundation for Statistical Computing, Vienna, Austria), with 95% confidence intervals estimated from 2000 bootstrap resamples and multiple testing correction by the Benjamini–Hochberg FDR method [30]. Alpha diversity indices (Shannon and Chao1) were calculated from rarefied tables in QIIME 2 (release 2021.8) and compared by ANOVA or Kruskal–Wallis tests, with Tukey or Dunn post hoc tests and FDR correction [31]. Beta diversity was assessed using Bray–Curtis dissimilarity on Hellinger-transformed matrices, ordinated by PCoA, and clustered by UPGMA in vegan v2.4-3. Group differences were tested by PERMANOVA (adonis, 9999 permutations), with dispersion checked by betadisper [32]. Community–environment relationships were modeled by redundancy analysis (RDA) in CANOCO 5.0 (999 permutations), and variance partitioning analysis (VPA) was conducted using vegan::varpart [33]. To assess the influence of shared taxa on between-sample similarity, pairwise counts of shared ASVs/OTUs were regressed against Bray–Curtis dissimilarity using linear models in R, and slopes were compared among trophic categories by ANCOVA [34]. Taxon niche breadth (Levins’ B) was calculated with spaa v0.2.2, classifying generalists as *B* > 2.0 and specialists as *B* < 1.5—their proportions were compared as an index of environmental sensitivity [35]. Core microbiome members were defined by prevalence thresholds using custom Python scripts executed in Python v3.8.10 (Python Software Foundation, Wilmington, DE, USA) [36]. Matrix correlations between community and environmental distance matrices were quantified by Mantel tests (Bray–Curtis vs. Euclidean/Gower, 9999 permutations) in vegan and visualized as network-style heatmaps with FDR-adjusted *p*-values [37].

### 2.6. Co-Occurrence Network Construction and Stability Evaluation

A random matrix theory (RMT)-based network inference pipeline was employed. To reduce sparsity and noise introduced by spurious correlations, the OTU/ASV tables were first filtered, retaining only taxa with a mean relative abundance > 0.01% and an occurrence frequency > 20% across samples. Spearman’s rank correlations were then calculated among the retained taxa, and significant correlations (*p* < 0.05) were used as candidate edges. The correlation threshold for network construction was automatically determined by the RMT algorithm to minimize random associations, resulting in |r| > 0.70 for bacterial networks and |r| > 0.66 for fungal networks. Network topological properties were computed in R v3.3.1 using the WGCNA and igraph packages, including the numbers of nodes and edges, average degree, network density, average path length, and modularity. Network visualization was performed in Gephi v0.9.2 using the Fruchterman–Reingold layout. Network stability was evaluated based on robustness and vulnerability metrics implemented in R v3.3.1 using the ggClusterNet package. Robustness was quantified by simulating random node removal (up to 50% of nodes) and tracking the decline in natural connectivity, with a slower decline indicating greater robustness. Vulnerability was defined as the maximum decrease in global network efficiency caused by removal of a single node, reflecting the network’s sensitivity to the loss of key taxa. Node topological roles were identified using within-module connectivity (Zi) and among-module connectivity (Pi), classifying nodes as peripherals (Zi < 2.5 and Pi < 0.62), module hubs (Zi > 2.5), connectors (Pi > 0.62), and network hubs (Zi > 2.5 and Pi > 0.62).

### 2.7. Statistical Analysis

Statistical analyses were conducted in IBM SPSS Statistics v22.0 and R v3.3.1, with diversity analyses in QIIME 2 (release 2021.8) and ordination analyses in CANOCO 5.0. Normality and homoscedasticity were checked using Shapiro–Wilk and Levene’s tests. Differences among habitats were tested using one-way ANOVA with Tukey’s HSD when assumptions were met; otherwise, Welch’s ANOVA with Games–Howell or Kruskal–Wallis with Dunn’s post hoc tests was applied (α = 0.05). Multiple testing was controlled using the Benjamini–Hochberg FDR correction. Alpha diversity (Shannon and Chao1) was calculated from rarefied tables in QIIME 2. Beta diversity was assessed using Bray–Curtis dissimilarity on Hellinger-transformed matrices, visualized by PCoA, and tested by PERMANOVA (adonis, 9999 permutations) with dispersion evaluated by betadisper (vegan). Community–environment relationships were examined by RDA (999 permutations) and variance partitioning analysis. Spearman correlations between environmental variables and dominant taxa were computed with bootstrap confidence intervals (2000 resamples). Mantel tests (9999 permutations) were used to relate community and environmental distance matrices.

## 3. Results

### 3.1. Soil Physicochemical Properties and Soil Enzyme Activities

The soil physicochemical properties and enzyme activities showed significant variations among the four soil types (Table 1). Soil water content (SWC) differed significantly across all sites, with PW (42.22%) being higher than TMW (18.40%), HHC (15.46%), and CF (13.80%; *p* < 0.05). All sites were alkaline, with pH ranging from 8.96 (CF) to 10.08 (HHC). The pH in HHC was significantly higher than in the other three sites. Nutrient levels were generally highest in the PW site. SOC in PW (35.03 g·kg^−1^) was greater than in TMW (26.22 g·kg^−1^), CF (20.98 g·kg^−1^), and HHC (8.02 g·kg^−1^; *p* < 0.05). Similarly, TN, TP, AN, and AP were highest in PW compared to the other sites (*p* < 0.05; Table 1). Soil enzyme activities followed a similar trend. URE (142.58 mg·g^−1^) and SUC (527.83 mg·g^−1^) activities were highest in PW. For ACP and CAT, activities were lower in HHC (77.63 mg·g^−1^ and 13.89 mg·g^−1^, respectively) than in PW, TMW, and CF, which did not differ significantly from one another.

### 3.2. Alpha Diversity of Bacterial and Fungal Communities Across Soil Types

The alpha diversity indices revealed significant differences in both bacterial and fungal communities among the four soil types (Table 2). For bacteria, CF exhibited the highest diversity and richness (Shannon = 6.32; Chao1 = 3323.11; Sobs = 2884.21), which were significantly greater than those in PW, TMW, and HHC (*p* < 0.05). HHC showed the lowest bacterial diversity, whereas PW and TMW were intermediate (Table 2). For fungi, the highest richness was also observed in CF (Chao1 = 436.63; Sobs = 397.17), significantly exceeding the other soil types (*p* < 0.05). In contrast, TMW showed the highest fungal Shannon diversity (3.88), which was significantly higher than that in HHC (2.61). PW and CF had intermediate fungal Shannon diversity (both 3.34), and PW showed the lowest fungal richness (Chao1 = 131.44; Sobs = 130.33).

### 3.3. Soil Microbial Community Composition Along Degradation Gradients

**Figure 2:**
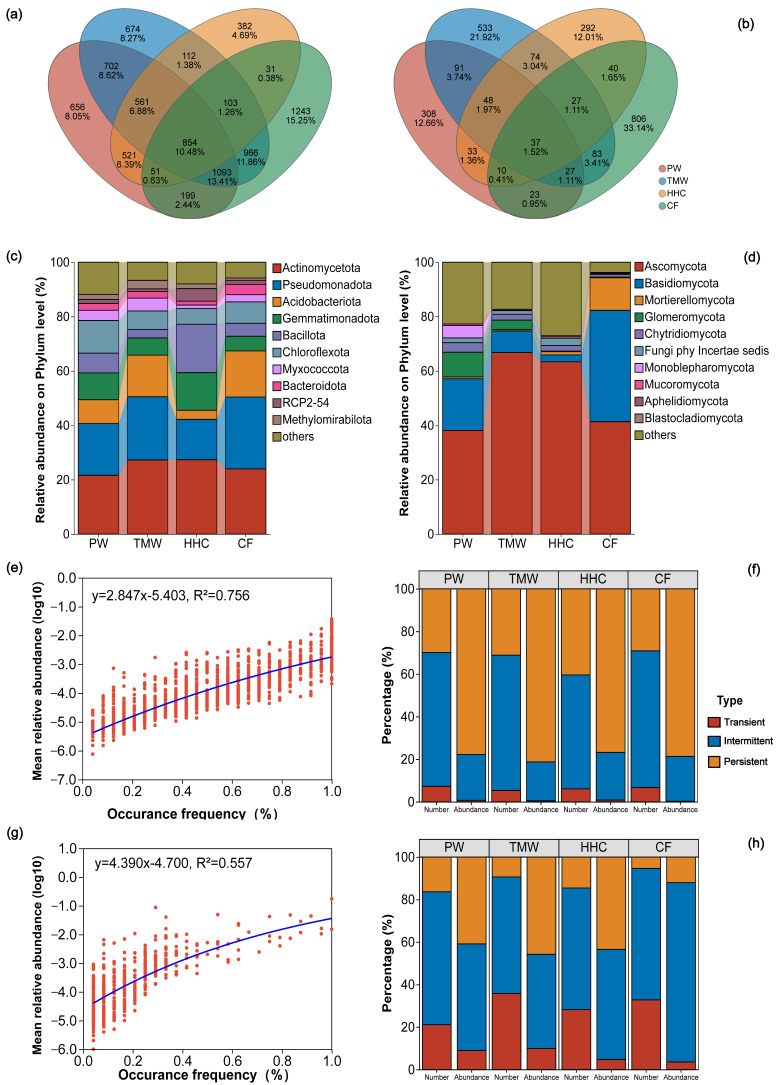
Taxonomic and ecological structure of soil microbial communities across a saline–alkaline degradation gradient. Venn diagrams of bacterial (**a**) and fungal (**b**) OTUs among four soil types. Relative abundances of bacterial (**c**) and fungal (**d**) phyla across soil types. Relationships between taxon prevalence and mean relative abundance for bacteria (**e**) and fungi (**g**), with R^2^ values indicated. Occupancy class composition (persistent, intermittent, and transient) of bacterial (**f**) and fungal (**h**) communities based on abundance and richness. Abbreviations: PW, pristine wetland; TMW, transitional meadow wetland; HHC, halophytic herbaceous community; CF, converted farmland.

Across all samples, 16,857 bacterial OTUs were detected, with 854 OTUs shared among the four soil types. TMW contained 5065 OTUs (674 unique), PW 4637 (656 unique), CF 4540 (1243 unique), and HHC 2615 (382 unique; Figure 2a and Figure S1a). For fungi, 3111 OTUs were detected in total, with 37 OTUs shared among soil types. CF contained 1053 OTUs (806 unique), TMW 920 (533 unique), PW 577 (308 unique), and HHC 561 (292 unique; Figure 2b and Figure S1b). At the phylum level, 55 bacterial phyla and 16 fungal phyla were observed. Dominant bacterial phyla included Actinomycetota, Pseudomonadota, Acidobacteriota, Gemmatimonadota, and Bacillota; for example, Acidobacteriota was higher in CF (16.95%) than HHC (3.31%), and Bacillota peaked in CF (17.83%; Figure 2c). Dominant fungal phyla were Ascomycota, Basidiomycota, Mortierellomycota, Glomeromycota, and Chytridiomycota, with Glomeromycota detected only in PW and TMW, Mortierellomycota enriched in CF (12.01%), and Monoblepharomycota detected only in PW (Figure 2d). Mean abundance was positively correlated with taxon prevalence, with a stronger relationship for bacteria (R^2^ = 0.756; Figure 2e) than fungi (R^2^ = 0.557; Figure 2g). Occupancy patterns differed between kingdoms (Figure 2f–h): bacterial communities were dominated by persistent OTUs in the abundance fraction, whereas intermittent OTUs contributed most to richness. Fungal communities showed a higher contribution of intermittent and transient OTUs, particularly in the richness fraction.

### 3.4. Beta Diversity and Taxonomic Turnover of Microbial Communities

**Figure 3:**
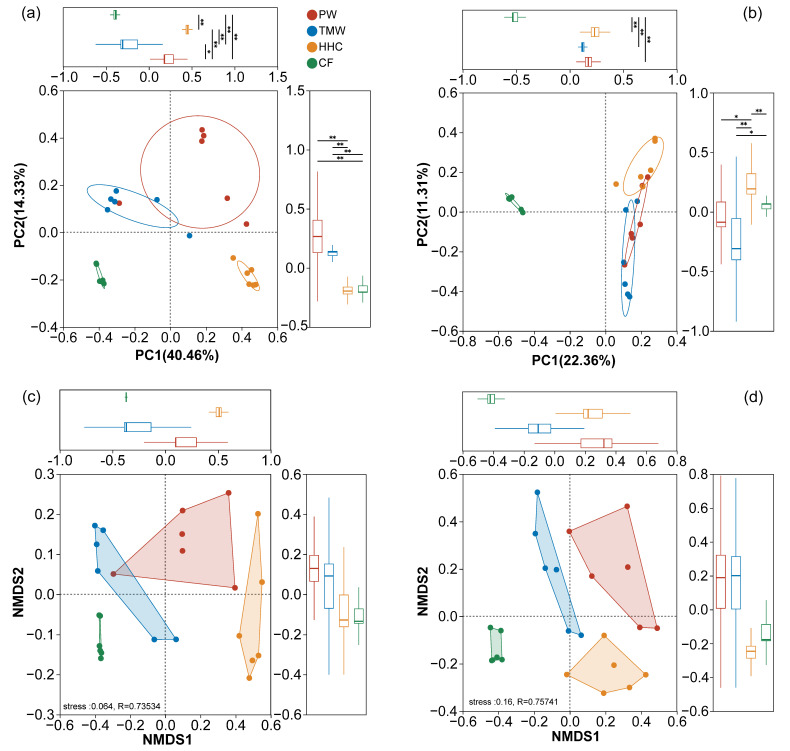
Ordination analysis of soil microbial beta diversity across degradation gradients. Principal coordinate analysis (PCoA) of bacterial (**a**) and fungal (**b**) communities—inset boxplots show statistical comparisons of PC1 scores. Non-metric multidimensional scaling (NMDS) of bacterial (**c**) and fungal (**d**) communities. Stress and ANOSIM R-values are indicated. Abbreviations: PW, pristine wetland; TMW, transitional meadow wetland; HHC, halophytic herbaceous community; CF, converted farmland. Asterisks in (**a**,**b**) denote significant differences in PC1 scores between groups (* *p* < 0.05 and ** *p* < 0.01).

PCoA resolved the compositional variation of bacterial and fungal communities (Figure 3a,b). For bacteria, PC1 explained 40.46% of the variance and PC2 explained 14.33%, cumulatively explaining 54.79% (Figure 3a). The PCoA plot showed clear separation along the PC1 axis, and PC1 scores of HHC and CF were significantly higher than those of PW and TMW (*p* < 0.01). For fungi, PC1 and PC2 together accounted for 33.67% of the variance (22.36% and 11.31%, respectively; Figure 3b). The fungal PCoA plot showed overlap among TMW, HHC, and CF, and PC1 scores indicated weak separation along this axis (no significant difference between TMW and HHC; Figure 3b). NMDS results were broadly consistent with these patterns (Figure 3c,d), with low-stress values for both bacteria (stress = 0.064) and fungi (stress = 0.057) and significant ANOSIM R values (bacteria: R = 0.73534; fungi: R = 0.75741).

### 3.5. Community Assembly Processes and Ecological Mechanisms

**Figure 4:**
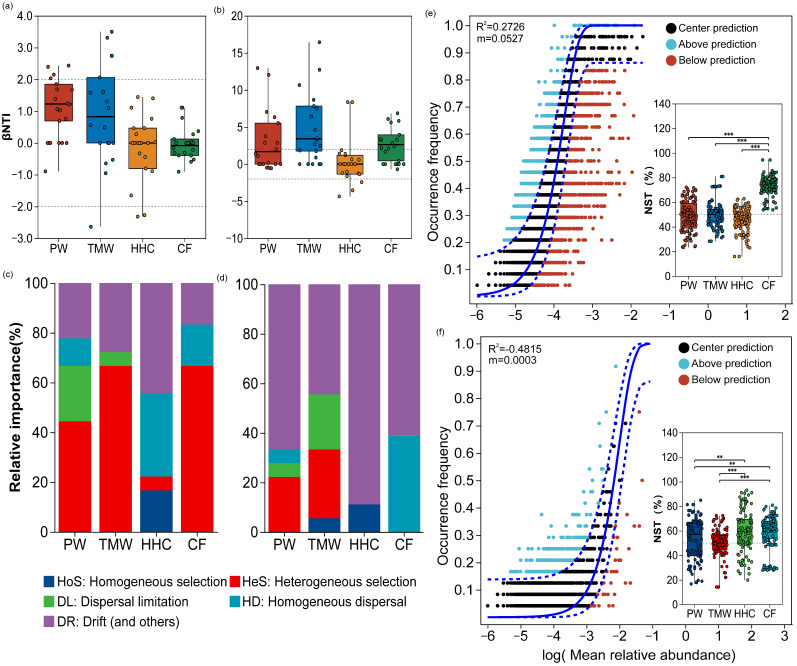
Ecological processes shaping microbial community assembly across land degradation gradients. β Nearest Taxon Index (βNTI) analyses for bacterial (**a**) and fungal (**b**) communities across soil types. Composite plots for bacteria (**c**) and fungi (**d**), showing the relative contributions of community assembly processes (qPEN; bar plots, left y-axis) and the normalized stochasticity ratio (NST; points, right y-axis). Neutral community model (NCM) fits for bacteria (**e**) and fungi (**f**), displaying observed occurrence frequency versus model predictions, with R^2^ and migration rate (m) values indicated. The solid line represents the NCM prediction and the blue dashed lines indicate the 95% confidence intervals. Abbreviations: PW, pristine wetland; TMW, transitional meadow wetland; HHC, halophytic herbaceous community; CF, converted farmland. ** *p* < 0.01 and *** *p* < 0.001.

Using βNTI thresholds (deterministic if |βNTI| > 2; stochastic if |βNTI| ≤ 2), within-type comparisons (PW–PW and TMW–TMW) were predominantly stochastic for both domains. However, between-type comparisons revealed divergent patterns (Table S1; Figure 4a,b). For bacteria, comparisons involving HHC (PW–HHC, TMW–HHC, and CF–HHC) and those including CF (CF–PW and CF–TMW) were most frequently assigned to the deterministic range. In contrast, fungal assembly was dominated by stochastic processes (|βNTI| ≤ 2) across nearly all between-type comparisons, including those involving HHC and CF. The PW–TMW contrast showed mixed assignments for both bacteria and fungi. Neutral community modeling showed a measurable fit for bacteria (R^2^ = 0.1292; Figure 4e), whereas the fit for fungi was poor (R^2^ = −0.4815; Figure 4f). Normalized stochasticity ratio (NST) results (Figure 4c,d) showed that bacterial NST exceeded 0.5 only in CF (mean = 0.735), while PW, TMW, and HHC were at or below 0.5. In contrast, fungal NST exceeded 0.5 across all soil types (PW = 0.527, TMW = 0.513, HHC = 0.597, and CF = 0.599).

### 3.6. Edaphic Controls on Microbial Composition, Niche Breadth, and Associations

**Figure 5:**
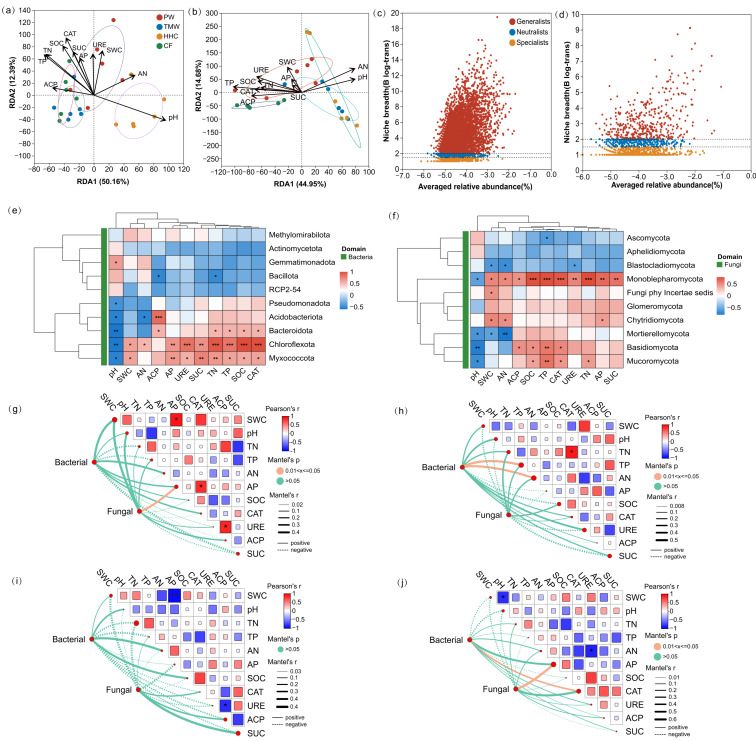
Environmental drivers of microbial community structure and associations. Redundancy analysis (RDA) biplots for bacterial (**a**) and fungal (**b**) communities. Scatterplots of taxon-level niche breadth for bacteria (**c**) and fungi (**d**). Correlation heatmaps for bacteria (**e**) and fungi (**f**) showing associations between taxa and edaphic/enzyme variables. Mantel test network heatmaps for PW (**g**), TMW (**h**), HHC (**i**), and CF (**j**), linking soil physicochemical and enzymatic indicators with microbial community dissimilarity. Abbreviations: PW, pristine wetland; TMW, transitional meadow wetland; HHC, halophytic herbaceous community; CF, converted farmland; SWC, soil water content; pH, soil pH; TN, total nitrogen; TP, total phosphorus; AN, available nitrogen; AP, available phosphorus; SOC, soil organic carbon; CAT, catalase activity; URE, urease activity; ACP, acid phosphatase activity; SUC, sucrase activity. Asterisks indicate statistically significant differences among vegetation types (* *p* < 0.05, ** *p* < 0.01, and *** *p* < 0.001).

Pooled redundancy analysis (RDA) constrained by edaphic and enzymatic variables resolved two canonical axes for both domains (Table S2). For bacteria (Figure 5a), RDA1 explained 50.16% and RDA2 12.39% of the constrained variance (cumulative 62.55%). Envfit retained pH, SOC, TN, TP, SWC, CAT, URE, SUC, and AN as significant vectors (all *p* ≤ 0.05), whereas AP and ACP were not significant at the pooled scale. For fungi (Figure 5b), RDA1 and RDA2 explained 44.95% and 14.68% of the constrained variance, and envfit identified pH, TP, and AN as significant vectors (all *p* ≤ 0.05). At the phylum level, pairwise heatmaps indicated that multiple bacterial and fungal groups were associated with moisture, nutrients, enzyme activities, and pH (Figure 5e,f). Niche-breadth analysis based on Levins’ metric showed a positive abundance–breadth trend in both domains, with bacteria dominated by generalists (66.39%) and fungi dominated by specialists (68.56%; Figure 5c,d). Site-specific Mantel tests (α = 0.05) identified limited significant links between community dissimilarity and individual edaphic distances (Figure 5g–j): PW showed a significant fungal association with AP, TMW showed significant bacterial associations with TP and AN, HHC showed no significant links for either domain, and CF showed a significant bacterial association with CAT and a significant fungal association with AP.

### 3.7. Divergent Responses of Bacterial and Fungal Co-Occurrence Networks to Wetland Degradation

**Figure 6:**
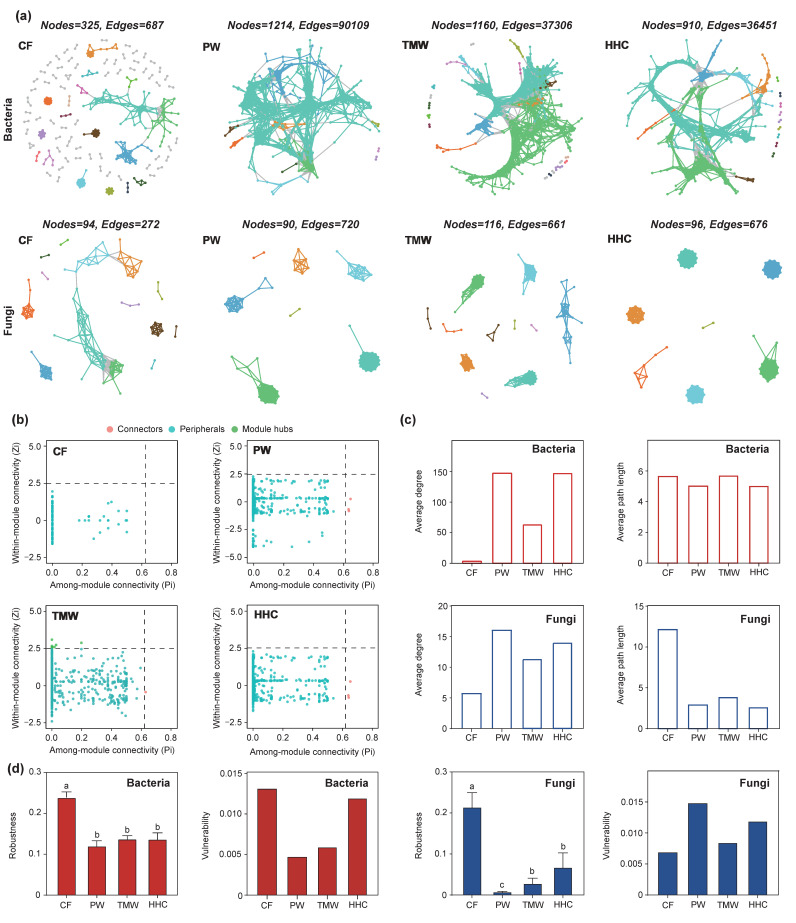
Co-occurrence network analysis of soil bacterial and fungal communities (**a**). Identification of key OTUs in bacterial network construction based on Zi-Pi analysis (**b**). Changes in network average degree and average path length (**c**). Evaluation of network stability using natural connectivity and vulnerability metrics (**d**). Different lowercase letters indicate significant differences among treatments according to Tukey’s HSD test (*p* < 0.05). Abbreviations: PW, pristine wetland; TMW, transitional meadow wetland; HHC, halophytic herbaceous community; CF, converted farmland.

Network analysis based on random matrix theory (RMT) revealed contrasting topological shifts and stability patterns of bacterial and fungal communities across the natural degradation sequence (PW–TMW–HHC) and the agricultural conversion state (CF; Figure 6). Bacterial networks were most connected in CF, with 1214 nodes and 90,109 edges and a high average degree (115.1). In HHC, the bacterial network was simplified, with edges decreasing to 36,451 (approximately 60% lower than CF) and the average degree dropping to ~78—the average path length increased from 2.8 (CF) to 3.8 (HHC; Figure 6c). In contrast, fungal networks showed higher connectivity under saline–alkali stress. The fungal network in CF was the simplest (94 nodes, 272 edges, average degree 5.79), whereas HHC showed higher connectivity (676 edges; average degree 14.08) and a shorter average path length (from 4.88 in CF to 1.06 in HHC; Figure 6c). Zi–Pi analysis indicated shifts in node topological roles, with fewer hubs/connectors in degraded bacterial networks but the emergence of highly connected nodes in degraded fungal networks (Figure 6b). Stability metrics were consistent with these structural changes (Figure 6d): the bacterial network in HHC showed higher vulnerability and a faster decline in natural connectivity under random node removal, whereas fungal networks exhibited lower vulnerability and higher robustness in degraded soils than bacterial networks.

## 4. Discussion

Wetland degradation in saline–alkali landscapes does not necessarily proceed as a single linear decline in ecosystem functioning. Our results instead support a threshold-driven transition in which hydrological change initiates divergent ecological outcomes [38]. Because we analyzed bulk soil rather than rhizosphere soil, our conclusions primarily reflect habitat-scale edaphic conditions in the 0–20 cm layer—rhizosphere effects warrant future paired rhizosphere and bulk soil sampling. The most prominent threshold signal was the sharp decrease in soil water content from the saturated wetland to the drier habitats, consistent with the view that hydrological collapse is a primary driver reshaping soil habitats and microbial activity during wetland degradation [39,40,41].

Following this hydrological shift, the system differentiated into two contrasting trajectories. The halophytic saline–alkali habitat represented a strong environmental filtration pathway, characterized by extreme alkalinity and marked nutrient depletion together with reduced enzyme activities, indicating weakened biochemical functioning under stress [42,43,44,45,46]. In contrast, the converted farmland reflected a management-shaped soil state in which drainage, tillage, and fertilization altered soil conditions and resource availability, preventing convergence toward the same extreme alkaline endpoint observed under natural degradation [47,48]. This divergence in soil context provides a mechanistic basis for interpreting the non-linear responses of microbial diversity and community structure across habitats.

Alpha diversity patterns were consistent with strong abiotic filtering under extreme saline–alkali stress but also highlighted that diversity responses differ by kingdom. The lowest bacterial diversity in the most stressed habitat aligns with deterministic exclusion under hyper-alkaline and nutrient-poor conditions, whereas the elevated bacterial diversity and richness in farmland indicate that agricultural conversion can generate a distinct niche landscape rather than a simple continuation of the natural degradation sequence [49,50,51,52,53]. Fungal diversity showed a different response, with richness highest in farmland but Shannon diversity peaking in the transitional habitat, suggesting that initial terrestrialization can increase habitat suitability and evenness before extreme chemical stress becomes dominant [54,55]. These contrasting patterns support the need to evaluate bacterial and fungal communities separately when interpreting degradation outcomes.

Community composition and turnover further indicate that bacteria and fungi are constrained by different aspects of habitat change. Bacterial phylum-level shifts tracked major edaphic gradients, consistent with clear ordination separation and with the strong role of soil geochemistry in structuring bacterial communities across saline–alkali environments and land-use types [56,57,58,59]. Fungal compositional changes were more consistent with hydrological and substrate transitions, including the loss of wetland-associated groups after drying and the enrichment of decomposer-associated taxa in farmland where crop residues provide abundant plant-derived inputs [60,61,62]. Together with the beta-diversity patterns, these results indicate that the post-threshold habitats represent distinct community configurations shaped by different dominant drivers rather than gradual drift along a single axis [63,64].

Null-model analyses provided direct evidence for kingdom-level divergence in assembly mechanisms. For bacteria, deterministic signals increased in comparisons involving the most stressed saline–alkali habitat and the converted farmland, indicating stronger selection under extreme stress and in the converted habitat [65,66,67]. In contrast, fungal assembly remained predominantly stochastic across habitats, consistent with weaker constraints from bulk soil variables and a larger role for dispersal limitation, ecological drift, and spatially heterogeneous substrates [68]. Edaphic association analyses supported these inferences. Bacterial community variation was strongly constrained by multiple soil properties and enzyme activities, whereas fungal communities were constrained by fewer bulk variables, consistent with niche-breadth patterns showing bacteria dominated by generalists and fungi dominated by specialists [69,70,71]. Under the most extreme stress, the weakening of site-specific community–environment links is consistent with a dominant constraint overriding finer-scale variation [72].

Co-occurrence networks added a structural perspective that complements the assembly results. Under extreme saline–alkali conditions, bacterial networks were simplified and exhibited higher vulnerability and a faster decline in natural connectivity during random node removal, consistent with reduced redundancy and increased structural fragility under strong environmental filtering [73]. In contrast, fungal networks showed tighter connectivity under stress and comparatively higher robustness. This pattern does not contradict predominantly stochastic fungal assembly, because stochastic turnover can coexist with tighter co-occurrence clustering when viable microhabitats and substrates become spatially compressed, promoting aggregation among stress-tolerant or functionally similar taxa [74,75]. Integrating network structure with assembly inference, therefore, strengthens the interpretation that bacteria are more tightly filtered by bulk soil geochemistry, whereas fungi reorganize associations under stress despite largely stochastic compositional turnover [76].

From a management perspective, the transitional meadow wetland represents a practical window for intervention because it occurs soon after the hydrological threshold shift but before the development of extreme alkaline conditions [77]. Maintaining or restoring hydrological connectivity and soil moisture at this stage may help prevent further divergence toward the severely filtered saline–alkali state [78]. For severely degraded saline–alkali habitats, restoration should prioritize reducing alkaline and salinity stress and rebuilding soil nutrient pools, coupled with vegetation recovery to enhance plant-derived inputs and microhabitat heterogeneity, thereby supporting the recovery and stability of both bacterial and fungal communities [79,80].

Overall, integrating soil properties and enzyme activities, diversity and compositional turnover, assembly processes, and network stability, saline–alkali wetland degradation is better described as pathway divergence following hydrological threshold loss rather than a linear decline [81]. One trajectory leads to an extreme filtration state with deterministic bacterial sorting, reduced biochemical potential, and fragile bacterial association networks, whereas fungal communities remain largely stochastic but can maintain comparatively robust co-occurrence structures under stress [82]. The other trajectory yields the converted farmland state with reorganized resource regimes, elevated bacterial diversity, and a restructured fungal community; however, such diversity gains should not be interpreted as inherently greater ecosystem stability, as they may depend on continued management [83]. Importantly, increased microbial diversity under agricultural conversion should not be equated with improved ecosystem health. Because 16S rRNA/ITS amplicon data have limited resolution for inferring human pathogenicity, we did not quantify the proportion of human-pathogenic microorganisms, and we suggest that future work apply targeted screening and functional approaches to evaluate potential health risks in managed wetlands. More broadly, the results highlight a fundamental decoupling of assembly rules between microbial kingdoms, with bacterial assembly tightly coupled to soil geochemistry and fungal assembly more strongly influenced by substrate heterogeneity and microhabitat structure [84,85,86].

## 5. Conclusions

This study shows that saline–alkali wetland degradation is not a simple linear decline but a threshold-triggered divergence into distinct ecological states. The sharp decrease in soil water content marks a key hydrological threshold, after which two contrasting trajectories emerge. The natural degradation trajectory is characterized by extreme alkalinity, nutrient depletion, suppressed enzyme activities, and a strengthened shift toward deterministic bacterial assembly. In contrast, agricultural conversion reshapes soil conditions and resource regimes through drainage, tillage, and fertilization, resulting in a soil state that differs from the naturally degraded saline–alkali endpoint. Bacterial communities were more tightly coupled to soil geochemistry, with stronger deterministic signals under both extreme stress and conversion conditions, whereas fungal communities remained predominantly stochastic and were comparatively weakly constrained by bulk soil properties. Co-occurrence networks further indicated that bacterial networks became simplified and more vulnerable under extreme saline–alkali stress, while fungal networks exhibited tighter co-occurrence structures and relatively higher robustness. Overall, these findings reveal a fundamental decoupling of assembly rules between bacteria and fungi and indicate that higher diversity under agricultural conversion does not necessarily imply greater intrinsic ecosystem stability. Two practical implications follow from these results. First, the transitional stage represents a priority window for intervention—rewetting and restoring hydrological connectivity at this stage may reduce the risk of further divergence toward the extremely filtered saline–alkali state. Second, restoration of severely degraded saline–alkali habitats should prioritize mitigating alkalinity and salinity stress and rebuilding soil nutrient pools, coupled with vegetation recovery to enhance substrate inputs and microhabitat heterogeneity, thereby promoting the recovery and stability of both bacterial and fungal communities.

## Figures and Tables

**Table 1:** Summary of soil physicochemical indices and enzyme activities by soil type.

Soil Properties	PW	TMW	HHC	CF
SWC (%)	42.22 ± 0.82 a	18.40 ± 1.22 b	15.46 ± 1.29 c	13.80 ± 0.53 d
pH	8.99 ± 0.08 b	9.19 ± 0.10 b	10.08 ± 0.25 a	8.96 ± 0.26 b
SOC (g·kg^−1^)	35.03 ± 2.79 a	26.22 ± 2.72 b	8.02 ± 1.07 d	20.98 ± 3.37 c
TN (g·kg^−1^)	6.30 ± 0.19 a	3.98 ± 0.71 b	0.75 ± 0.09 c	3.68 ± 0.71 b
TP (g·kg^−1^)	2.92 ± 0.05 a	1.99 ± 0.21 c	1.40 ± 0.05 d	2.61 ± 0.04 b
AN (mg·kg^−1^)	436.12 ± 21.52 a	368.15 ± 22.32 b	376.77 ± 6.26 b	259.70 ± 18.67 c
AP (mg·kg^−1^)	59.70 ± 2.67 a	52.33 ± 3.14 b	45.37 ± 3.47 c	47.27 ± 4.51 c
CAT (mg·g^−1^)	24.32 ± 5.01 a	18.36 ± 0.61 a	13.89 ± 0.27 b	18.89 ± 1.53 a
URE (mg·g^−1^)	142.58 ± 33.14 a	89.38 ± 10.64 b	74.39 ± 12.48 b	88.97 ± 18.48 b
ACP (mg·g^−1^)	173.77 ± 29.17 a	164.77 ± 24.62 a	77.63 ± 4.48 b	157.78 ± 10.12 a
SUC (mg·g^−1^)	527.83 ± 54.26 a	343.67 ± 77.03 b	99.83 ± 15.43 d	174.67 ± 40.28 c

Note: All data are expressed as the mean ± standard error of six replicates (*n* = 6). Different lowercase letters indicate significant differences among treatments according to Tukey’s HSD test (*p* < 0.05). Abbreviations: PW, pristine wetland; TMW, transitional meadow wetland; HHC, halophytic herbaceous community; CF, converted farmland; SWC, soil water content; pH, soil pH; TN, total nitrogen; TP, total phosphorus; AN, available nitrogen; AP, available phosphorus; SOC, soil organic carbon; CAT, catalase activity; URE, urease activity; ACP, acid phosphatase activity; SUC, sucrase activity.

**Table 2:** Soil bacterial and fungal alpha diversity across the degradation gradient.

Types	Bacteria			Fungi		
	Shannon	Chao 1	Sobs	Shannon	Chao 1	Sobs
PW	5.83 ± 0.23 b	2330.12 ± 457.26 b	1982.54 ± 378.14 b	3.34 ± 0.72 ab	131.44 ± 71.44 b	130.33 ± 70.31 b
TMW	6.03 ± 0.12 b	2664.45 ± 261.61 b	2253 ± 204.79 b	3.88 ± 0.43 a	237.6 ± 103.81 b	233.67 ± 100.35 b
HHC	4.93± 0.36 c	1296.35 ± 463.75 c	1104.57 ± 336.88 c	2.61 ± 0.29 b	137.66 ± 83.35 b	132 ± 76.64 b
CF	6.32 ± 0.08 a	3323.11 ± 103.63 a	2884.21 ± 115.05 a	3.34 ± 0.57 ab	436.63 ± 80.02 a	397.17 ± 59.22 a

Note: All data are expressed as the mean ± standard error of six replicates (*n* = 6). Different lowercase letters indicate significant differences among treatments according to Tukey’s HSD test (*p* < 0.05). Abbreviations: PW, pristine wetland; TMW, transitional meadow wetland; HHC, halophytic herbaceous community; CF, converted farmland.

## Data Availability

Data will be provided as requested.

## Supplementary Materials

The following supporting information can be downloaded at: https://www.mdpi.com/article/10.3390/biology15010061/s1. Figure S1: Upset plots of bacterial (**a**) and fungal (**b**) OTU overlap among soil types. Table S1: Comparison of environmental sensitivity of soil microbial communities. Table S2: Comparison of environmental sensitivity of soil microbial communities.

## Abbreviations

The following abbreviations are used in this manuscript:

ACP	Acid phosphatase activity
AMF	Arbuscular mycorrhizal fungi
AN	Alkali-hydrolyzable nitrogen
ANOSIM	Analysis of similarities
AP	Available phosphorus
βNTI	Beta Nearest Taxon Index
CAT	Catalase activity
CF	Converted farmland
EC	Electrical conductivity
HHC	Halophytic herbaceous community
NMDS	Non-metric multidimensional scaling
NST	Normalized stochasticity ratio
OTU	Operational taxonomic unit
PCoA	Principal coordinates analysis
PLS-DA	Partial least squares discriminant analysis
PW	Pristine wetland
RDA	Redundancy analysis
SOC	Soil organic carbon
SRA	Sequence Read Archive
SUC	Sucrase activity
SWC	Soil water content
TMW	Transitional meadow wetland
TN	Total nitrogen
TP	Total phosphorus
URE	Urease activity
