# Supplementary material for: Divergent Assembly of Bacteria and Fungi During Saline–Alkali Wetland Degradation
Source: Biology (Basel). 2025 Dec 29;15(1):61. doi: 10.3390/biology15010061 (PMC12784867; doi:10.3390/biology15010061)
Supplement: Supplementary file 1 [file biology-15-00061-s001.zip › Supplementary material.pdf]

Table S1. Comparison of Environmental Sensitivity of Soil Microbial Communities

Types	Group	Abundance % -Transient	Abundance% -intermittent	Abundance% -Persistent	Number（pct.%） -Transient	Number（pct.%） -intermittent	Number（pct.%） -Persistent
Bacteria	PW	0.72%	21.52%	77.76%	272（7.29%）	2344（62.82%）	1115（29.88%）
	TMW	0.48%	18.27%	81.25%	194（5.38%）	2288（63.49%）	1122（31.13%）
	HHC	0.83%	22.41%	76.75%	142（6.09%）	1246（53.48%）	942（40.43%）
	CF	0.27%	21.08%	78.64%	264（6.71%）	2527（64.2%）	1145（29.09%）
Fungi	PW	8.91%	50.21%	40.88%	58（21.09%）	172（62.55%）	45（16.36%）
	TMW	9.93%	44.31%	45.77%	195（35.78%）	299（54.86%）	51（9.36%）
	HHC	4.68%	51.89%	43.43%	93（28.18%）	189（57.27%）	48（14.55%）
	CF	3.56%	84.38%	12.06%	331（32.77%）	625（61.88%）	54（5.35%）

Table S2. Data related to the Redundancy Analysis (RDA)

**Types**	**Items**	**CCA1**	**CCA2**	**r^2**	***P* value**
	SWC	0.190	-0.982	0.796	0.001
	pH	0.840	0.542	0.833	0.001
	TN	-0.457	-0.889	0.859	0.001
	TP	-0.683	-0.731	0.641	0.001
	AN	0.626	-0.779	0.849	0.001
Bacteria	AP	-0.070	-0.998	0.675	0.001
	SOC	-0.344	-0.939	0.702	0.001
	CAT	-0.316	-0.949	0.590	0.001
	URE	0.021	-1.000	0.397	0.003
	ACP	-0.811	-0.585	0.255	0.045
	SUC	-0.171	-0.985	0.557	0.002
	SWC	0.590	0.808	0.913	0.001
	pH	0.457	-0.890	0.840	0.001
	TN	0.048	0.999	0.877	0.001
	TP	-0.283	0.959	0.914	0.001
Fungi	AN	0.961	0.275	0.935	0.001
	AP	0.513	0.858	0.687	0.001
	SOC	0.055	0.999	0.855	0.001
	CAT	0.088	0.996	0.572	0.001
	URE	0.386	0.923	0.587	0.001
