# Supplementary figures and images for: Divergent Assembly of Bacteria and Fungi During Saline–Alkali Wetland Degradation
Source: Biology (Basel). 2025 Dec 29;15(1):61. doi: 10.3390/biology15010061 (PMC12784867; doi:10.3390/biology15010061)

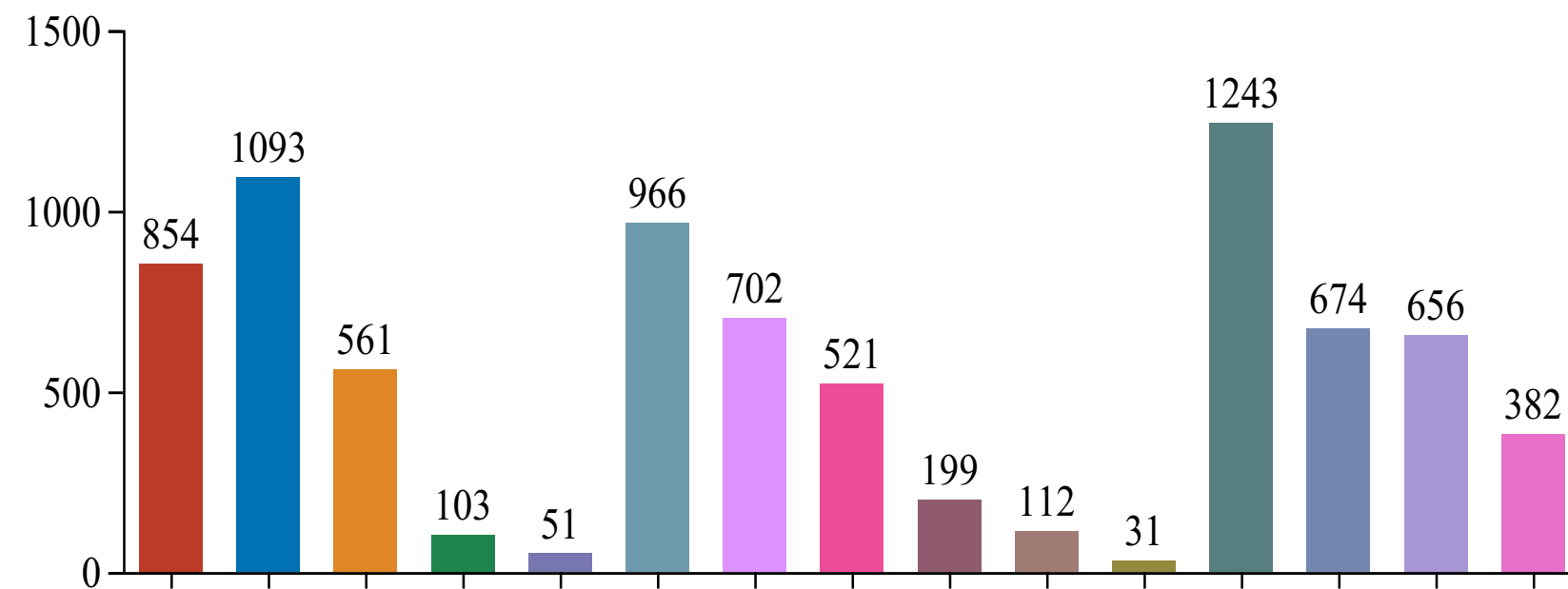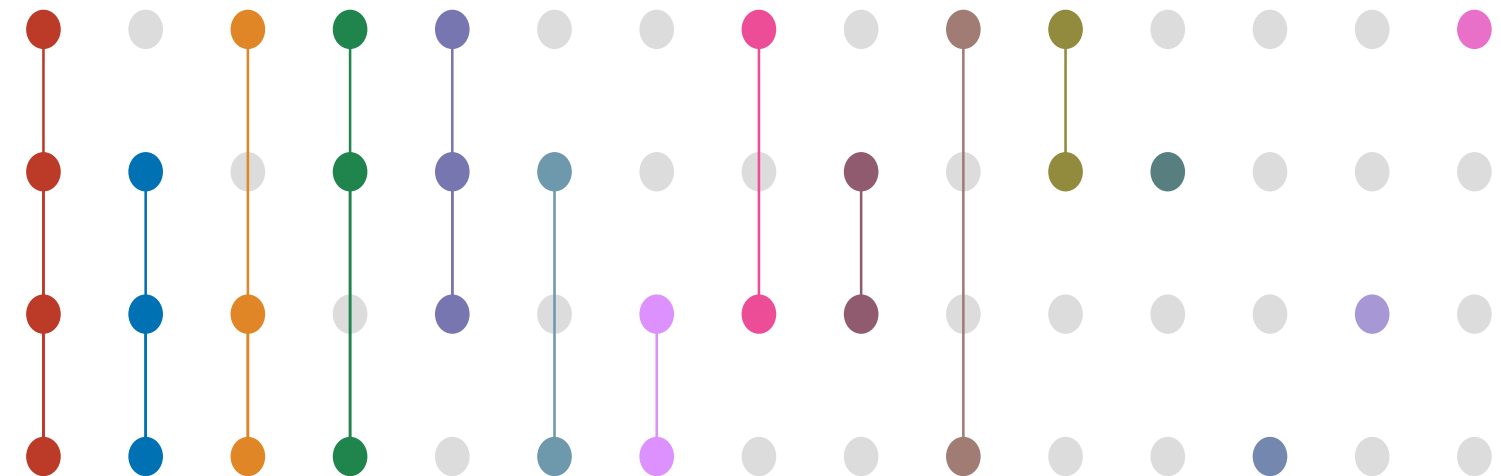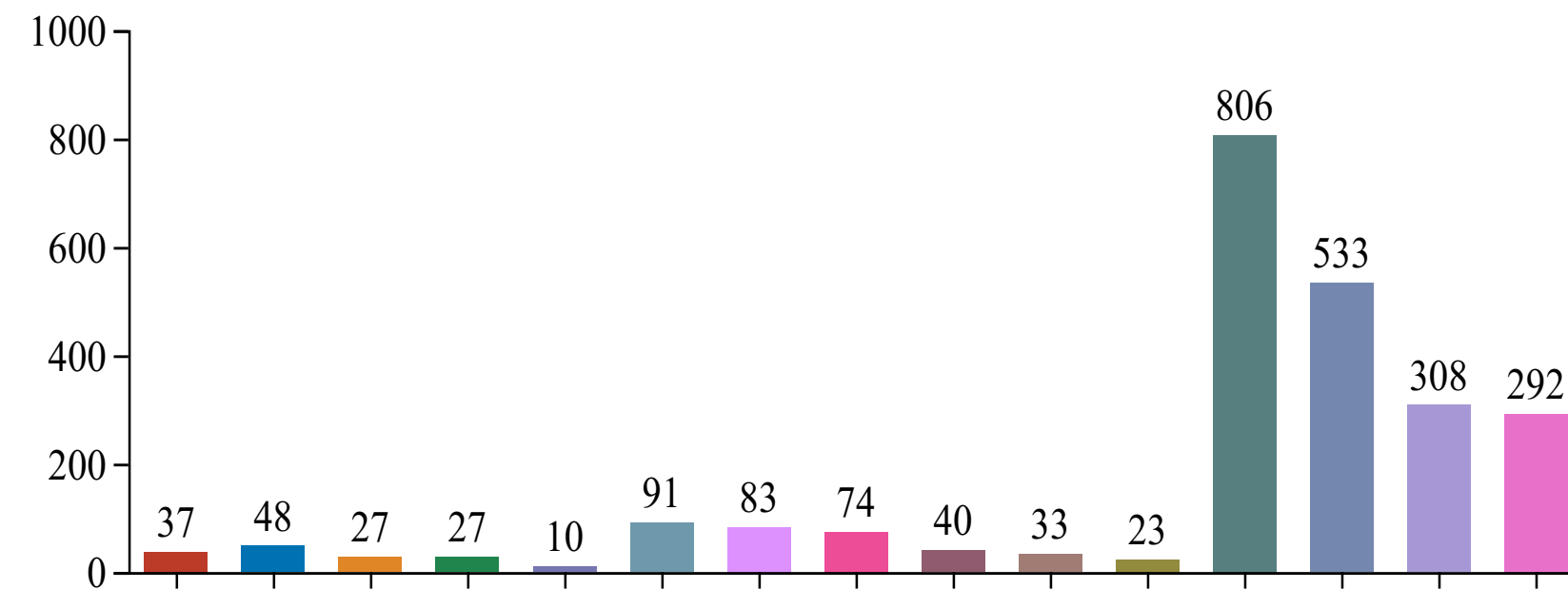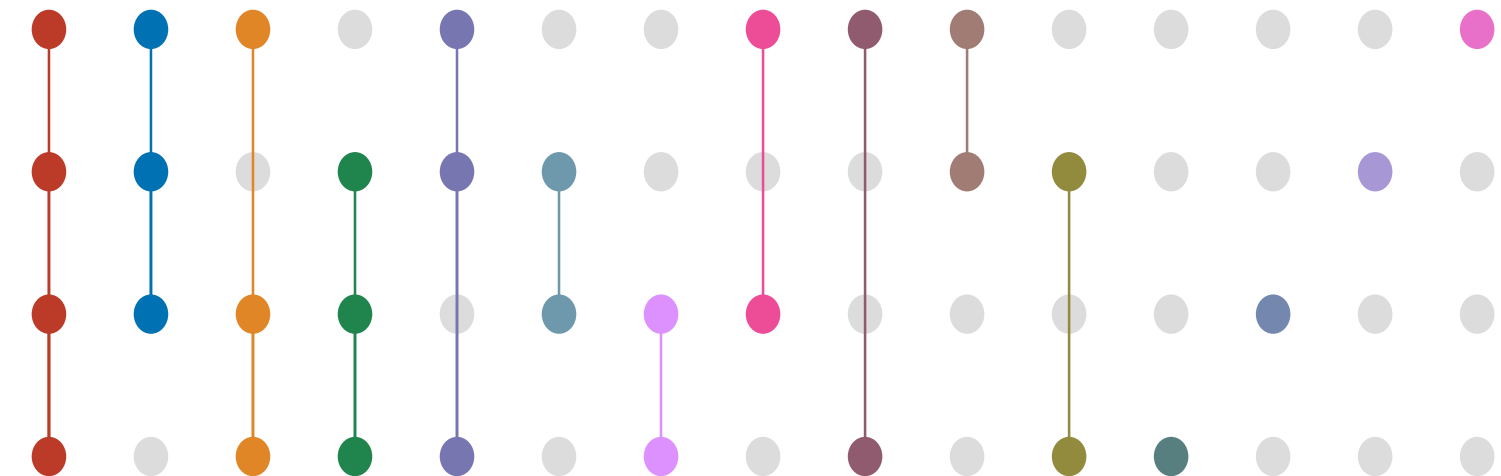

Supplement: Supplementary file 1 [file biology-15-00061-s001.zip › Figure S1.pdf]
